# Supplementary material for: Are carbon emissions trading and green financial instruments synergistic? -Comprehensive quantitative research based on content analysis
Source: PLoS One. 2024 Mar 7;19(3):e0298601. doi: 10.1371/journal.pone.0298601 (PMC10919601; doi:10.1371/journal.pone.0298601)
Supplement: S1 Data — (ZIP) [file pone.0298601.s001.zip › Data/地级市Z_F工作报告.zip!/╔╜╢1⁄2/╚╒╒╒╩╨/└┤╫╘╣1⁄2╓┌║┼í╛┬φ┐╦╩2╛▌═°í┐_2018.docx]

各位代表：

　　现在，我代表市人民政府向大会报告工作，请予审议，并请市政协各位委员提出意见。

一、2017年工作回顾

　　过去的一年，是我市发展进程中具有重要意义的一年。一年来，我们坚持以习近平新时代中国特色社会主义思想为指导，在省委、省政府的坚强领导下，按照中共日照市委部署要求，全面贯彻“一、三、五”总体发展思路，加快推进新旧动能转换，打赢了一场场攻城拔寨的硬仗，取得了一系列重要成果。

　　——经济运行持续向好。主要指标增幅跃居全省前列，跑出了“日照加速度”。预计全年实现地区生产总值2020亿元，一般公共预算收入141.3亿元，分别增长9.2%、12.3%；固定资产投资1765亿元、规模以上工业增加值610亿元，分别增长10.5%、10.3%；社会消费品零售总额726亿元，增长10%；进出口总值913亿元、增长11.3%，其中出口348亿元、增长24.7%。三次产业比例调整为7.5：48.5：44。一批“四新”“四化”重大项目加快推进，新旧动能转换初见成效。山钢日照精品钢基地完成投资337亿元，引进多项世界首创技术，生产装备、环保水平全球领先，一期一步试车投产，一期二步全面开工。日钢转型升级成效显著，独家引进全球最先进的ESP生产线5条、酸洗和镀锌线5条，连续两年位列全国钢铁企业竞争力第一梯队。投资40亿元的中兴汽车开工建设，整车生产资质和三个目录公告落户日照。投资120亿元的海汇新能源汽车开工建设。长城汽车整车项目签署框架协议。国际财富中心、白鹭湾科技金融小镇建成运营。东方太阳城、莒国古城、太古飞机维修、空客模拟机研发生产、派沃泰三工厂、科创中心、万达广场等一批大项目相继开工。日照港东煤南移、集装箱码头改造工程开工。213个市重点项目完成投资585亿元。园区配套设施完成投资251亿元。招引产业项目511个，到位资金339亿元。粮食总产89万吨，特色种植面积150万亩，创建国家级海洋牧场3处、省级海洋牧场8处。全年接待游客4434万人次，旅游消费总额360亿元。完成港口货物吞吐量4.07亿吨，机场旅客吞吐量73.7万人次。

　　——改革开放深入推进。重点领域改革取得突破性进展，发展活力进一步增强。调整理顺高新区管理体制，创建国家高新区工作进入科技部考察名单。设立空港经济开发区管理机构。园区职员制、薪酬制改革全面铺开。“放管服”改革不断深化，新下放审批事项97项，创新实行“容缺受理”“并联审批”，在全省率先实现“三十五证合一”。

市场主体达到21.46万户。国资国企改革成效明显，市属国企资产总额2350亿元，融资390亿元，投资230亿元。43家市属国企二级企业完成混改。综合行政执法体制改革基本完成。1327个村完成农村集体产权制度改革。莒县、东港区分别被确定为国家和省级农村集体产权制度改革试点县。全面推进中小学校长职级制和教师“县管校聘”改革，1337名校长教师参与城乡交流轮岗。启动公立医院法人治理结构改革。主动融入“一带一路”，对外开放迈出新步伐。在澳大利亚成功举办中澳产业园投资说明会。澳斯亚德帆艇、盛安东水产加工示范园等8个项目落户中澳产业园。综合保税区申建进入海关总署审批程序。岚桥集团巴拿马玛岛港扩建工程开工建设。集装箱粮食指定口岸、进境木材检疫处理区建成启用，肉类和冰鲜水产品指定口岸建成待批，6个新建泊位正式开放。实际利用外资42.3亿元，境外投资14亿元。日照港改革整顿成效显著，新开通日照至欧洲、至中亚、至越南集装箱国际班列，至澳大利亚、至中东、至东南亚、至日本关东关西航线，成功实现对巴基斯坦卡西姆港管理输出，全年实现利润10.1亿元，创建港以来最好水平。

　　——城乡面貌大为改观。城市“颜值”与“气质”同步改善，人居环境、公共服务、社会文明风尚显著提升。以全国第二名的成绩跻身全国文明城市，顺利通过“国家卫生城市”复审，成功创建“国家森林城市”“国家生态保护与建设典型示范区”。莒县、五莲县创建为“国家园林县城”。交通基础设施拉起主框架。青日连铁路正在铺轨，年内将与济青高铁同步建成通车，鲁南高铁明年建成。日照西综合客运站即将建成，日照综合客运站提升改造全面开工。机场高速建成通车，潍日高速今年10月份建成通车，岚罗高速加快推进。山海路、北京北路、迎宾路改造一二期建成通车。206国道、222省道改建工程竣工。石臼港区北京路上海路畅通工程、深圳路东延工程竣工。204国道绕城段改建、厦门路建设工程开工。打通莒州路等19条断头路。改善提升县乡公路14条，300个省、市贫困村全部实现通公路、通客车。城市功能和居住环境不断提升。持续推进创城突出问题综合整治，新老城区“两重天”得到彻底改变，礼让斑马线、光盘行动等文明行为蔚然成风，网格长、街长、楼长、店长“四长”机制初步建立。拆迁城中村112个，整治老旧小区55个，建设棚改安置房57224套。治理建成区违法建设259.7万平方米。规划建设国际会展中心、科技馆、大剧院、未来馆、文博中心、星级酒店群等一批大项目。新建市民公园、兴海公园、体育公园、莒州公园等休闲公园10处、170万平方米。改扩建海曲公园16万平方米。建设“口袋公园”17处、9万平方米。绿化提升北京路等12条道路。亮化主城区道路9条、建筑186座。建设综合管廊12条。日照至岚山天然气长输管道开工建设。建成山东路、青岛路公交专用道。完成城乡客运班线公交化改造。新开通城乡公交线路67条，实现镇镇通公交。新投放新能源公交车100辆，新增自行车站点100个、投放公共自行车2000辆。巨峰镇、刘官庄镇分别入选全国、全省第二批特色小镇。建设美丽乡村示范片7个，省级示范村20个。完成农村改厕18.8万户。林水会战、蓝天保卫战、清清河流行动、矿山综合整治等重大举措成效显著。全年造林15.6万亩，林木覆盖率42%。建立市县乡村四级“河长”体系。完成水利工程1680项。城市日供水保障能力76万方。农村集中供水覆盖率达70%。治理河流152公里，省控以上断面实现达标。治理整顿矿山178处、关停39处。“蓝繁”天数310天。

　　——民生事业不断进步。一大批惠民举措落地实施，人民群众获得感明显增强。全市财政民生支出187 . 2亿元，占一般公共预算支出的80.6%，增长13.7%。城镇、农村居民人均可支配收入分别达到30700元、14500元，分别增长8.5%、8.8%。城乡低保分别提高到每人每月520元、340元。城乡特困人员救助供养标准分别提高到每人每年不低于12036元、9012元。完成全民参保登记。实现跨省异地就医联网结算。发放创业担保贷款4.2亿元、就业创业补贴1.35亿元，带动创业就业1.63万人。城镇新增就业4.14万人。企业最低工资标准提高到每人每月1640元。投入11.8亿元，新改扩建学校（幼儿园）113所、65.6万平方米。山东外国语职业学院升本工作完成省级考察评审。日照海事专修学院升格为高职专科院校。市特殊教育学校建成启用。全面两孩政策稳步实施，出生人口性别比大幅下降。市医院综合体、中医院综合体、心脏病医院等重点项目加快推进。建设全民健身工程、智慧健身场馆470处。奥林匹克水上运动小镇被确定为全国首批试点。成功举办马拉松、啦啦操、太极拳、机器人等国际性赛事，承办全国单车耐力赛、全国百城校园足球联赛等20多项重大赛事。举办太阳文化国际研讨会、第八届中国少儿合唱节、马拉松年度峰会等重大活动。开展“喜迎十九大·唱响新日照”群众性系列文化活动4000余次。两城镇考古遗址公园列入第三批国家考古遗址公园立项名单。

　　——社会治理更加有效。民主法治、公平正义、安定有序的社会局面不断巩固。安全生产、生态环保、脱贫攻坚、社会稳定、金融风险防控攻坚战成效明显。安全生产事故起数、死亡人数实现“双下降”，关停整顿企业50家，立案729起。办结中央、省、市环保督察案件710件，整改企业1721家，立案处罚189家。在全省率先实现低保线和扶贫线“两线合一”，完成减贫1.6万人，贫困户危房改造5263户。基本完成不良贷款处置和贸易融资风险化解。累计处置不良贷款522亿元，立案183起。排查稳定风险隐患7960件，化解7386件。投入专项资金4019万元，解决退役人员就业、生活等困难。圆满完成十九大安保维稳任务。“七五”普法扎实推进。刑事警情同比下降11.7%。信访形势持续好转。成功捧得全国社会治安综合治理最高奖“长安杯”。查处作风问题152起、处理302人。纪检监察机关立案760件、处分672人。查处群众身边腐败问题146起、处理182人。日照港集团系列案件立案120人，23人已被“双开”。推出“电视问政”4期。自觉接受人大、政协监督。向市人大常委会提交法规案3件，在全省率先出台城市管理条例、物业管理条例。支持人民政协参政议政。支持各民主党派、工商联、无党派人士参与重大决策民主协商。办复人大代表建议182件，政协提案344件，办复率、面办率、满意和基本满意率100%。国防动员、民族宗教、外事侨务、对台事务、妇女儿童、残疾人、老龄、慈善、红十字、档案、气象、人防、仲裁、打私、史志、防震减灾等工作都取得了新成绩。

　　各位代表！征途充满艰辛，奋斗铸就辉煌。过去的一年，是我市攻坚克难、创新发展的一年，是转型提速、蓄势积能的一年。经过全市人民的不懈奋斗，经济社会各个领域都发生了深刻变化，日照发展翻开了崭新的一页。一年来的成绩，是中共日照市委团结带领全市人民共同奋斗的结果，是市人大、市政协和社会各界有效监督、大力支持的结果。我代表市人民政府，向全市人民，向各民主党派、各人民团体和各界人士，向驻日照人民解放军指战员、武警官兵、公安干警，向关心支持日照发展的海内外朋友，表示衷心感谢！

　　我们也清醒看到，日照发展不平衡不充分问题还比较突出，优势和潜力远远没有发挥出来。城乡发展不平衡，县域经济和园区发展薄弱。经济规模偏小，发展质量不高，“四新”经济发展缓慢，集聚人气的能力弱，新旧动能转换任务艰巨。农业现代化水平不高，“新六产”发展层次低。工业产业链条短，创新能力弱，产品附加值低。总部经济、楼宇经济、会展经济等业态刚刚起步，现代服务业发展滞后。人才、科技、对外开放短板突出。生态、港口、海洋等优势挖掘不够，“港产城海”融合发展不充分。水、大气、土壤污染防治任重道远，资源环境承载压力大。民生领域欠账较多，教育、医疗、康养、文化等优质公共服务供给不足。城市功能不完善，精细化管理水平不高。一些单位懒政、怠政和不作为、乱作为现象仍然存在。这些问题，必须采取有力措施，认真加以解决。

二、2018年总体思路和预期目标

　　2018年是贯彻党的十九大精神的开局之年，是改革开放40周年，是决胜全面建成小康社会、实施“十三五”规划承上启下的关键一年。做好今年的工作，意义重大，影响深远。政府工作总的要求是：深入贯彻落实党的十九大精神，以习近平新时代中国特色社会主义思想为指导，坚持稳中求进工作总基调，坚持新发展理念，按照高质量发展要求，全面落实“一、三、五”总体发展思路，加快推进新旧动能转换，坚定不移实施“突破园区、聚力招引”头号工程，打好防范化解重大风险、精准脱贫、污染防治三大攻坚战，更加注重经济转型升级，更加注重城乡统筹协调，更加注重增进民生福祉，壮大发展规模，集聚城市人气，着力解决发展不平衡不充分问题，建设美丽富饶、生态宜居、充满活力的现代化城市。

　　主要预期目标是：地区生产总值增长9%左右，一般公共预算收入增长10%左右；规模以上工业增加值增长11%左右，固定资产投资增长11%左右；社会消费品零售总额增长10%左右；进出口总值保持平稳增长；城镇和农村居民人均可支配收入分别增长8.2%和8.5%左右；常住人口城镇化率达到60.5%；城镇登记失业率控制在3.5%以内；居民消费价格指数涨幅控制在3%左右；完成省下达节能减排降碳等约束性目标。

　　各位代表！中国特色社会主义进入了新时代，我国社会主要矛盾发生了历史性变化，我国经济已转向高质量发展阶段。全省新一轮大开放、大发展全面起跑。这对政府工作提出了许多新挑战、新要求。机遇稍纵即逝，竞争更加激烈。我们必须主动适应这种新变化，准确把握新时代发展规律，加快工作指导转变，最大限度挖掘优势、激发潜力、补齐短板，努力实现更高质量、更有效率、更加公平、更可持续的发展！

　　各位代表！市委十三届二次全会描绘了日照未来发展的宏伟蓝图，开启了建设现代化城市的新征程。实现“追赶”“摘帽”和“双增双突破”目标，日照有优势、有潜力，全市人民有能力、有担当。今天的日照，“航空梦”“整车梦”已经实现，“高铁时代”即将到来，“港产城海”融合发展的新格局正在加快形成，日照的发展进入了从蓄势积能到发力超越的新阶段。站在新的历史起点上，我们必须担当起时代赋予的使命，保持战略定力，坚定发展信心，持续用力、久久为功，扎扎实实做好政府工作，让经济更加繁荣，让城市更加美丽，让人民更加幸福，努力创造无愧于时代、无愧于人民、无愧于历史的新业绩！

　　三、挖掘增长潜力，厚植发展优势，构建具有日照特色的现代化经济体系

　　紧扣我国社会主要矛盾变化，按照高质量发展要求，坚持以供给侧结构性改革为主线，在“破”“立”“降”上下功夫，加快推动质量变革、效率变革、动力变革，增强经济创新力和竞争力。

　　（一）坚定不移推进“突破园区、聚力招引”头号工程，加快新旧动能转换。坚持世界眼光、国际标准、日照特色，聚焦以“四新”促“四化”，实施海洋经济产业基地等10个重大专项，推进中兴汽车、科创中心等100个市重点项目。高标准建设日照先进钢铁制造基地。落实园区“二次规划”“多规合一”，促进产业集聚，推动园区特色发展。完善园区基础设施，加快公共服务平台建设，提高园区承载力。完成园区基础设施投入200亿元以上。坚持精准招商，深度对接世界500强、央企和民营强企，主攻大项目招商、产业链招商、资本市场招商，严把安全、环保、效益关，提高招商成功率和回报率。积极推进“人才兴市”战略，实施日照籍人士回乡创业创新工程。新引进过5000万元产业项目230个以上，过10亿元项目20个以上，实际到位资金300亿元以上。引进高层次创业创新团队70个、创新人才420名，院士、千人计划专家16人以上。日照高新区创建为国家级。加快岚山开发区、空港开发区、山海天度假区升级步伐。

　　（二）做好建链强链补链文章，振兴工业经济。深入实施“工业强市”战略，壮大主导产业集群，激发民营企业活力，推动工业提质扩量。坚持抓龙头、铸链条、建集群，实施钢铁及配套、石油化工、汽车及零部件等主导产业提升计划，建材、能源、纺织服装等传统产业转型计划，高端装备、生物医药、信息技术、新材料等新兴产业倍增计划。山钢日照精品钢基地一期二步完成主体工程，日钢4号ESP生产线建成投产、5号线全面开工，加快建设钢铁配套产业园，钢铁产业产值突破1000亿元。中兴汽车建成投产，支持五征汽车、海汇新能源汽车发展。推动优势零部件企业高端化发展，提高本地配套率。坚持“支线运输、通航运营、航空制造”三位一体，发展航空产业，争创国家通用航空产业综合示范区。深入开展化工产业安全生产转型升级专项行动，推动危化品企业“进区入园”，发展高端化工产业。加快粮油食品产业升级步伐，培育市级重点企业20家。推动木材产业园提档升级，打造木材精深加工产业集群。推进亚太森博溶解浆等项目建设，拉长浆纸包装印刷产业链条。加快海尔硬创智慧产业园、装配式建筑产业基地建设。实施机器人自动化装备改造等百项重点工业项目。推进20家企业实施机器换人、100家企业上云、30个两化融合项目。

　　毫不动摇鼓励、支持、引导非公有制经济发展，激发各类市场主体活力。打造公平竞争营商环境，有效破解融资难、融资贵问题。全面清理涉企收费。开展“红顶中介”专项治理。实施小微企业治理结构和产业结构“双升”战略，促进民营企业向“专精特新”发展。鼓励企业开展跨行业、跨区域兼并重组。建立龙头企业、上市企业、科技型企业培育库。开展质量提升行动，推进质量强市和商标品牌战略，鼓励企业开展境外商标注册、国际认证和专利申请，推动企业从产品、价格竞争向质量、品牌竞争转变。激发和保护企业家精神，建设高素质企业家队伍。新增规模以上工业企业100家以上，民营企业5000家以上，民营经济增加值增长15%。

　　（三）推动旅游业向中高端迈进，大力发展现代服务业。深入实施“旅游富市”战略，以创建国家全域旅游示范区为抓手，做大做强旅游业。开展全市旅游资源普查。培育康体养生、体育运动、海洋体验、文化休闲等新业态。创建国家中医药健康旅游示范区。加快建设东方太阳城、莒国古城、金帆山海汇、中华国医坛等116个重点旅游项目，完成投资160亿元以上。东夷小镇、海洋馆与四季花鸟园投入运营。发展奥林匹克水上运动小镇、智慧康养小镇、太极小镇、白鹭湾艺游小镇等一批特色旅游小镇。高度重视城市形象宣传推介。建设智慧旅游城市，完善集散中心、休闲街区、度假酒店等服务设施。规范旅游标识。推进“厕所革命”，加强旅游市场综合整治，推动乡村游提质增效。举办国际太极拳、水上运动会、国际马拉松、中国体操节“四大赛事”，承办第二十四届省运会自行车、皮划艇比赛，办好省第八届全民健身运动会开闭幕式，打造活力之城、运动之城、健康之城。全年接待游客4800万人次，旅游消费总额400亿元以上。

　　加快服务业转型升级。推动生产性服务业向专业化、高端化延伸，生活性服务业向精细化、品质化提升。大力发展总部经济、楼宇经济、会展经济，加快奥林匹克公园西岸综合开发，打造中央活力区。丰富金融业态，培育基金、互联网金融等新兴服务业，促进多层次资本市场健康发展。白鹭湾科技金融小镇市场主体新增500家、达到1200家以上。国际财富中心注册公司新增200家、达到300家以上。壮大临港物流业，建设区域性国际航运物流中心。推动大宗商品交易中心创新发展。建成传化公路港物流园。推进天泽冷链物流基地与京东合作，打造全国冷链物流示范城市。全社会物流总额增长10%以上。大力发展电子商务、工业设计、商务服务等新业态。完善商业网点布点规划，建设一批特色商圈、特色街区。推进智慧便利店进社区、智能快件箱进楼宇工程，加快标准化菜市场建设，打造“15分钟便民消费圈”。

　　（四）发挥港口和海洋资源优势，建设海洋强市。强化经略海洋意识，坚持以港口为龙头、产业为核心、城市为载体、海洋为支撑，做好“港产城海”融合发展文章。

　　发挥港口引领城市发展的龙头作用，提升港口要素集聚和资源配置能力，打造国际一流大港强港。开展港口发展战略研究，优化港口发展规划。巩固日照港改革整顿成果，提升港口经营管理水平和盈利能力，促进港口转型升级、创新发展，建设诚信、智慧、高效、绿色港口。规划建设临港商务区，培育发展邮轮经济，实现港口与临港工业、现代服务业、集疏运体系协同发展。投资50亿元，实施东煤南移、LNG码头、岚山港区深水航道二期等15项重点工程。实行岚山港危化品作业区封闭运行。加快推进中岚铁路、岚罗高速、疏港路西延等项目建设，构建高效便捷的集疏运体系。实施新增船舶扶持和外流船舶回归计划。进一步开拓国际航线，加快实施内陆无水港、国际物流园等项目，打造“一带一路”综合性枢纽港、示范港。全年完成港口货物吞吐量4.2亿吨，集装箱吞吐量400万标箱。

　　坚持陆海统筹，发展向海经济，向海洋要财富、要空间。开展海洋发展战略研究，制定海洋强市战略规划。争创国家海洋经济发展示范区。加快“海上粮仓”、海洋牧场建设。落实深远洋渔业发展扶持政策，支持远洋渔业发展。加快推进黄海冷水团等项目建设，新发展海洋牧场10万亩，高标准建设国家海洋牧场示范区。提升海水养殖业，壮大海产品加工业。做优休闲渔业。发展海洋生物医药产业。建设海洋装备研发制造基地，做大海洋高端装备制造业。加快沿海渔港经济区建设。落实海洋生态红线制度，推进近岸海域综合治理，开展蓝色海湾整治行动。实施“退港还海”“退渔还湿”“南红北柳”等海岸带修复工程。建立海洋大数据中心，依法科学用海，提高海洋资源开发利用水平。

　　（五）深化改革开放创新，增强内生动力。聚焦重点领域和关键环节，大力推进改革开放创新，使市场在资源配置中起决定性作用，更好发挥政府作用。

　　靠改革破解难题。深化“放管服”改革。开展“证照分离”改革试点，推进“多证合一”，加强“双随机一公开”监管。深化综合行政执法体制改革。实行跨部门跨领域综合执法，推进部门“一支队伍执法”，构建“综合执法+专业执法”新体制。深化国资国企改革。优化国有资本投向，做大做强主业。完善国企考核分配制度。市属国企资产总额达到2600亿元。深化财政改革。加强政府债务风险防控。深化投融资体制改革。增强金融支持实体经济能力。盘活基础设施存量资产。规范政府购买服务和PPP模式，探索推进资产证券化。推广公建民营等模式，加快公共服务领域市场化改革。全年投融资600亿元以上。

　　靠开放拓展空间。深入实施“开放活市”战略，突出“一带一路”沿线城市，加强与欧美、澳大利亚和周边国家、地区的交流合作；依托瓦日铁路、鲁南高铁等大通道，扩大与中西部经济板块的开放合作；主动对接周边城市，在规划衔接、产业合作、基础设施互联互通等领域，建立广泛的城市合作伙伴关系，形成陆海内外联动、东西双向互济的开放格局。依托海上丝绸之路，积极融入国际产能合作。加快综合保税区、中澳产业园等开放平台建设。完成海港口岸扩大开放验收，肉类及冰鲜水产品指定口岸投入运营。争取空港口岸临时开放，开通国际包机航班。推进机场扩建工程和通用机场建设。

　　靠创新强化支撑。争创国家创新型试点城市。鼓励企业加大研发投入，实施市级重大科技专项，全社会研发经费支出占GDP比重达到1.6%以上。实施高新技术企业培育计划，新增高新技术企业50家以上，高新技术产业产值增长20%以上。完成科创中心主体工程建设。创建日照钢铁、深远海养殖等省级技术创新中心。省级以上孵化器、众创空间等创新平台，实现区县全覆盖。争创省级农高区。加强与国内外高校、科研院所合作。推动黄海技术交易市场建设。培育知识产权优势企业。争创国家知识产权示范城市。引进转化先进技术成果100项以上。

　　四、加快乡村振兴，提升城市品质，打造城乡经济社会融合发展新格局

　　坚持乡村振兴、县域突破、城市提升“三位一体”，把城市和农村作为一个整体来谋划，建立健全城乡融合发展体制机制，重塑城乡关系，促进城乡共同繁荣、全域协调发展。

　　（一）实施乡村振兴战略。坚持农业农村优先发展，把农村和城市摆在平等地位，按照产业兴旺、生态宜居、乡风文明、治理有效、生活富裕的总要求，制定实施乡村振兴战略规划，努力让农业成为有奔头的产业，让农民成为有吸引力的职业，让农村成为安居乐业的美丽家园。

　　深化农村土地制度改革，完善承包地“三权”分置办法，探索开展土地经营权质押。推进莒县国家级、东港区省级农村集体产权制度改革试点。完成农村集体资产清产核资。盘活农业农村资源，让农民分享集体经济增值收益。规范农村产权流转交易市场，增加农民财产性和工资性收入。坚持质量兴农、绿色兴农，转变农业发展方式，发展特色优势产业，加快推进农业现代化。大力发展农业“新六产”，实施国家级农村一二三产业融合试点项目。加快现代农业产业园建设。发展多种形式适度规模经营，走产业化、企业化、市场化发展路子，提高农业综合效益。支持有条件的乡村建设田园综合体。新发展市级以上农民合作社、示范家庭农场60家。加强生态循环农业示范基地建设。落实粮食安全责任制。完善农产品质量安全追溯体系。实施商标品牌战略，大力培育地理标志商标和农产品商标。实施日照绿茶品质品牌提升工程。加强新型职业农民教育培训、认定管理和政策扶持。加快美丽乡村建设，建成市级示范片区8个、省级示范村20个。

　　（二）突破县域经济。深入推进新一轮县域经济提升行动，加强县域规划指导，推动要素配置、公共服务、基础设施互联互通，发挥比较优势，实现错位发展，打造各具特色的“板块经济”。支持东港区重点发展现代服务业和高新技术产业，加快空港开发区、太阳文化度假区开发建设；岚山区重点发展钢铁、高端石化等临港产业，加快中澳产业园、钢铁配套产业园等园区建设；莒县重点发展新能源汽车、机械制造、高端化工、文化旅游和现代农业，争创国家级绿化模范县、国家级生态县；五莲县重点发展全域旅游、生态产业和汽车及农用装备产业，创建国家全域旅游示范区。全省县域经济发展中，“两区”上升2个以上位次，“两县”上升3个以上位次。稳步推进乡改镇、镇改街道。加快农业转移人口市民化。坚持产业“特而强”、功能“聚而合”、形态“小而美”、机制“新而活”，引导特色小镇健康发展，培育巨峰茶香小镇、潮河白鹭湾科技金融小镇、后村航空小镇、刘官庄云塑小镇等一批特色小镇。

　　推动基础设施向农村延伸，促进基本公共服务均等化。深入推进城乡交通、公交、环卫、供气、供水、供电“六个一体化”。实施“幸福公路”三年行动计划，新建改造农村公路项目28个、1100公里。完善莒北莲西路网体系。实施村庄道路“户户通”工程。新开通公交线路21条，完成城乡公交化改造线路137条。全面推行城乡生活垃圾分类制度。开展镇村污水集中治理。实施“气化日照”工程，实现天然气镇镇通。加快农村公共供水管理体制改革，实施农村饮水巩固提升行动，农村集中供水覆盖率达到80%，改善18万农村居民饮水安全条件。投资6.3亿元，新建续建输变电工程16处。统筹推进城乡教育、卫生、文化、体育、康养等公共服务一体化。

　　（三）提升城市规划建设管理水平。强化系统思维，推进生产、生活、生态“三生融合”发展，巩固全国文明城市、国家森林城市成果，创建国家生态园林城市，持续提升城市环境质量和人民生活质量。

　　坚持高起点规划。注重以人为本、尊重自然、传承历史、绿色低碳，加强城市发展战略研究，为城市远景发展预留弹性空间。创新城市规划编制理念，推进智慧城市、海绵城市、旅游城市建设。开展“国家城市设计试点市”工作。规划一批国际化城市标志性景观，加强城市建筑群、公共空间的精致化设计。统筹“港产城海”布局，实施城市总体设计、城市“双修”等16项规划编制。统筹推进县域、镇村规划。

　　坚持高标准建设。实施道路畅通工程。新改建德州路等城市道路25条。推进迎宾路快捷路改造、合村路建设、204国道改建、厦门路等工程建设。实施管网配套工程。建设综合管廊3条，完善提升10条。实施北京路、兴海路等17条道路、99.5公里雨污分流。推进工业供水管道和水厂提升改造，开工建设城西水厂，新建改造配水管网37.3公里，完成老旧小区户表改造5万户。扩建改造污水污泥处理厂。实施城市功能提升工程。加快日照综合客运站、国际会展中心、科技馆、大剧院、未来馆等项目建设。完成美术馆、博物馆新馆建设。续建市民公园、体育公园，新增绿地2000亩。实施“300米见绿、500米见园”工程，新建石臼中心公园、沭河公园等88处城市公园绿地，新增绿地8000亩。加强重点人防工程建设，强化地下空间开发利用。推进莒县沭东新城23个重点工程建设。实施五莲县城东、城北片区开发。

　　坚持精细化管理。严格落实城市管理条例、物业管理条例，用“绣花功夫”管好城市。坚持重心下移、属地管理，理顺城乡社区管理服务体制，完善社区配套用房和公共服务设施，健全网格化管理体系。规范城市标识标牌。建设临沂路、昭阳路等6条公交专用道。新建市区公交候车亭100处、乡村公交候车亭350处。投放新能源公交车100辆、公共自行车4000辆。建立智慧交通一卡通平台。实施交通信号灯配时优化工程。推进黄海一路、海曲路等8条道路周边建筑亮化。开展城市违法建设和道路“六清”治理。整治老旧小区67个、167万平方米。改造城中村66个、房屋1.78万处，开工建设棚改安置房8.6万套。

　　争创国家生态园林城市。坚持生态优先、绿色发展，统筹城市发展与生态保护，增强城市可持续发展能力。开展国土绿化行动，丰富生物多样性，提升城市生态功能。强化城市绿线管控，打造园林绿化品牌。增强城市承载力和吸引力，提升城市生活品质，集聚城市人气，促进产城融合。强化政策引导、制度创新、公众参与，形成共建共治共享的良好氛围。力争通过三年努力，成功创建国家生态园林城市。

　　（四）推进生态文明建设。深入实施“生态立市”战略，放大生态优势，努力让日照的天更蓝、山更绿、水更清、环境更优美。

　　把林水会战融入乡村振兴战略、美丽乡村建设、扶贫攻坚行动，不断丰富内涵、提质增效。坚持建管并重，创新推行市场化、物业化、专业化管护机制。继续实施“八大绿化工程”“六大兴水工程”，投资44.7亿元，新增造林15万亩，建设各类水利工程1722项。以204国道环城线、222省道等13条道路为重点，实施通道连绿，打造生态廊道。倡导全民植树造林。新建省级森林镇村17个，绿化提升村庄207个。加强傅疃河口、两城河口、绣针河口等湿地公园保护修复。开展清清河流行动，严格落实河长制，实施河道治理工程47项、494公里。除险加固小型水库43座，发展节水灌溉15万亩，治理水土流失61平方公里。城市日供水保障能力达到80万方。

　　实行最严格的环境保护制度，严守生态保护红线。完善主体功能区制度和生态补偿机制。加大环保督察力度，严惩环境违法违规行为。开展污染源普查。严格落实“水十条”“气十条”“土十条”，深入实施污染防治攻坚行动，着力解决突出环境问题。加强水资源管理保护，严守用水总量、用水效率、水功能区限制纳污“三条红线”，建设节水型社会。健全饮用水源地保护机制，加大重点流域水质改善力度，确保省控以上断面全面达标。坚决打赢蓝天保卫战，“蓝繁”天数保持在300天以上。建设日照水库环库湿地和乡村振兴战略试验区。创建全省山水林田湖草系统治理示范市。严格执行环评审批负面清单，完善网格化环境监管体系。推广畜禽清洁生产，废弃物综合利用率达到80%。推进绿色发展，开展创建节约型机关、绿色家庭、绿色学校、绿色社区和绿色出行等行动。

　　五、坚守民生底线，提高保障和改善民生水平，织密扎牢覆盖“人的一生”民生保障网

　　坚持以人民为中心的发展思想，多谋民生之利、多解民生之忧，

在幼有所育、学有所教、劳有所得、病有所医、老有所养、住有所居、弱有所扶上，努力满足人民群众美好生活需要。

　　（一）坚决打赢脱贫攻坚战。坚持专项扶贫、行业扶贫、社会扶贫“三位一体”，深入推进“十大精准扶贫行动”，完善贫困群众增收和社会保障、救助政策，基本完成脱贫任务。突出抓好莒北、莲西、港西、岚西北区域扶贫，统筹推进扶贫开发和乡村振兴战略，提升基础设施、产业发展和基本公共服务水平。注重扶贫与扶志、扶智相结合，实施贫困村提升工程，完善扶贫长效机制。坚持精准扶贫、精准脱贫，坚决防止数字脱贫、虚假脱贫，做到脱真贫、真脱贫。

　　（二）办好公平优质教育。优先发展教育事业，推进教育公平，建设教育强市。着力解决中小学生课外负担重、“大班额”等突出问题，解决好婴幼儿照护和儿童早期教育服务问题。实施普惠性幼儿园扩增工程，采取公建民营、民建公助等模式，新改扩建普惠性幼儿园100所，实现“一社区一普惠性幼儿园”。高度重视农村义务教育，在财政投入、师资力量等方面向农村倾斜，实现城乡校长教师交流轮岗制度化常态化，推动城乡义务教育一体化发展。普及高中阶段教育，毛入学率达到98%以上。实施第二期特殊教育提升计划。支持社会力量兴办教育，新建一批民办学校（幼儿园）。深化中小学校长职级制、教师县管校聘、考试招生制度等综合改革。加强产教融合、校企合作，完善现代职业教育体系，培养高素质技术技能人才。支持高等职业院校创建国家优质、示范性院校，支持中等职业学校加快发展。为驻日照高校创造良好环境，促进校地双方共同发展。加强师德师风建设，培养高素质教师队伍，倡导全社会尊师重教。办好继续教育，建设学习型社会。

　　（三）促进就业创业。落实新一轮就业创业扶持政策，以大众创业、万众创新拓展就业空间。加大就业援助力度，强化社会兜底保障，做好失业人员、贫困人口等重点群体就业工作。完善众创空间、见习基地等平台，打造一批大学生创业园、创业街区，吸引更多大学生来日照就业创业。鼓励创业带动就业，支持农民工等人员返乡创业创新。引导支持农村妇女等群体，发展种植养殖、手工制作、电子商务、庭院经济等产业项目。扩大职业技能培训规模，开展企业订单、培训机构列单、劳动者选单、政府买单“四单”培训，促进就业提质扩量。健全职业培训、就业服务、劳动维权“三位一体”农民工服务机制。完成职业技能培训1万人，新增城镇就业2.88万人。

　　（四）完善社会保障体系。实施全民参保计划，完善基本养老保险和基本医疗保险制度，提高社会保险综合覆盖率。增强失业保险、工伤保险、大病保险保障能力。完善长期医疗护理保险政策。通过政府购买服务，全面推行民生综合保险。城乡低保分别提高到每人每月550元、395元，城乡特困人员救助供养标准分别提高到每人每年不低于12720元、10116元。开展居家和社区养老服务改革。实现乡镇敬老院公办民营改革全覆盖。新增养老床位1000张，每千名老人床位达到37张。加强农村留守儿童、妇女、老人关爱服务，做好困境儿童、残疾人等特殊群体保障工作。支持慈善事业发展。

　　（五）建设健康日照。深化医药卫生体制改革，健全现代医院管理制度。完成城乡卫生服务体系布局规划。建成启用日照心脏病医院，加快市医院综合体、中医院综合体建设。积极推进港口医院与北大医疗合作项目。加快基层医疗卫生机构标准化建设。推进医联体建设，深化医保支付方式改革，促进优质医疗资源上下贯通，构建分级诊疗服务体系。加强全科医生队伍建设。做实家庭医生签约服务，贫困人口家庭、计划生育特殊家庭、残疾人家庭签约率100%。建设全民健康信息平台。发展健康产业，推进医养结合，鼓励社会资金进入养老、医疗等领域。开展卫计系统行风整顿，加强医德医风建设。建设全民健身工程200处，开展全国幼儿体育、运动处方试点。倡导健康文明生活方式，推动全民健身与全民健康深度融合。创建全民运动健身模范市。

　　（六）推动文化繁荣发展。践行社会主义核心价值观，扎实推进“四德工程”，深化群众性精神文明创建活动。完善公共文化服务体系，打造“活力日照、乐享文化”系列品牌。推进城市书房建设。深化文化体制改革。创建文化强省建设先进市。加大文化产业扶持力度，推进文化创意产业园等重点项目建设。打造“日照·太阳之城”城市品牌。举办太阳文化研讨会、文化产业博览会、文化惠民消费季等活动。挖掘弘扬太阳文化、海洋文化、莒文化等历史文化，提升沂蒙画派、农民画、黑陶、绿茶等文化品牌。加强文物和非物质文化遗产保护。繁荣文艺创作，推出更多富有时代特色的文艺精品。推进移风易俗，树立文明乡风，营造向上向善的良好社会风尚。

　　（七）保持社会和谐稳定。巩固“长安杯”成果，深化平安日照、法治日照建设，推动由“平安”迈向“长安”。深入推进“七五”普法。完善“一村一法律顾问”“一村一警务助理”制度，建设覆盖城乡的公共法律服务体系。加强综治中心标准化建设。推进社区矫正基地建设。高度重视信访工作。健全矛盾排查预警和调处化解机制，及时回应群众诉求。强化公安基层基础建设。实施“天网工程”“雪亮工程”，完善社会治安防控体系。做好重大活动安保工作。开展打黑除恶、打霸治痞、打击逃废金融债务等专项行动。健全“1+4”多重预防体系，坚决遏制重特大安全事故发生。实施餐饮质量安全提升工程，加强食品药品安全监管。争创国家食品安全示范城市。加强社会信用体系建设。支持法院、检察院依法履职。支持驻军、武警部队建设。争创全国双拥模范城“六连冠”。做好援疆和扶贫协作工作。

六、切实加强政府自身建设

　　打铁必须自身硬。面对新形势新任务，我们必须坚持党对一切工作的领导，牢固树立“四个意识”，用宽肩膀担当，靠真本领成事，不忘初心、牢记使命，恪尽职守、永不懈怠，不断提升政府履职能力和服务水平，努力建设人民满意政府。

　　始终坚持依法行政。强化法治思维，严格按照法定权限行使权力、履行职责，建设法治政府。落实重大事项决策法定程序，完善专家论证、风险评估等制度，发挥法律顾问和社会智库作用，推动行政决策科学化、民主化、法治化。全面推进政务公开，让权力始终在阳光下运行。自觉接受监督，做好人大建议、政协提案办理工作，积极听取社会各界人士意见。健全政府新闻发言人和信息发布制度，保障群众知情权、参与权、监督权。

　　始终坚持为民执政。把人民利益摆在至高无上的地位，让发展成果更多惠及人民群众。以群众满意为衡量政府工作的标尺，将更多精力放到服务基层、服务群众上来，用干部的辛苦指数换取人民的幸福指数。坚持问政于民、问需于民、问计于民，用好“电视问政”、政务服务热线等平台，畅通政府与公众互动渠道。完善与企业、商会协会联系沟通机制，守底线、讲规矩，当好“店小二”，构建“亲”“清”新型政商关系。

　　始终坚持高效施政。大兴学习调研之风，找准短板弱项，解决实际问题。练就“八种本领”，培养专业能力，弘扬专业精神，推动政府治理能力现代化。整合共享政务信息资源，建设全市统一的政务服务平台。落实“容缺受理”“并联审批”等制度，优化工作流程，以“最多跑一次”倒逼简政放权。倡树勇于担当、立说立行的工作作风，发扬苦干实干和“钉钉子”精神，坚决整肃庸政、懒政、怠政行为。完善正向激励和容错纠错机制，坚决杜绝“劣币驱逐良币”现象。

　　始终坚持从严治政。落实全面从严治党要求，深入推进党风廉政建设和反腐败斗争，做到干部清正、政府清廉、政治清明。认真贯彻中央八项规定精神，以永远在路上的坚韧，坚决纠正“四风”，严肃查处形式主义、官僚主义问题。始终把纪律和规矩挺在前面，让干部知敬畏、存戒惧、守底线，习惯在受监督和约束的环境中工作生活。发挥审计保障监督作用。强化督查问责。持之以恒正风肃纪。严厉查处群众身边腐败问题。运用好监督执纪“四种形态”，抓早抓小、防微杜渐，构建不敢腐、不能腐、不想腐的有效机制，营造风清气正良好政治生态。

　　各位代表！新时代赋予新使命，新起点开启新征程。让我们紧密团结在以习近平同志为核心的党中央周围，深入贯彻习近平新时代中国特色社会主义思想，在中共日照市委的坚强领导下，锐意进取、苦干实干、善作善成，为加快建成美丽富饶、生态宜居、充满活力的现代化城市而努力奋斗！
